# Extrapulmonary Infections Associated with Nontuberculous Mycobacteria in Immunocompetent Persons

**DOI:** 10.3201/eid1509.081259

**Published:** 2009-09

**Authors:** Claudio Piersimoni, Claudio Scarparo

**Affiliations:** United Hospitals, Ancona, Italy (C. Piersimoni); Santa Maria della Misericordia University Hospital, Udine, Italy (C. Scarparo)

**Keywords:** Nontuberculous mycobacteria, immunocompetent subject, extrapulmonary infections, lymphadenitis, osteoarticular infections, skin and soft tissue infections, laboratory diagnosis, synopsis

## Abstract

Over the past several years, the prevalence of human disease caused by nontuberculous mycobacteria (NTM) has increased. Whether the increase in cases is real or whether more cases are being recognized remains unclear. Despite a considerable increase in knowledge about NTM infections, they still represent a diagnostic and therapeutic challenge for several reasons: 1) pathogenic isolates may be indistinguishable from contaminant or saprophytic isolates; 2) timely and reliable identification of isolates may depend on proper communication between clinicians and laboratory staff; 3) lack of standardized susceptibility testing makes adoption of tailored therapies unrealistic; and 4) lack of treatment guidelines exposes patients to toxic drugs and disappointing outcomes. Laboratory research and multicenter controlled trials are needed to improve diagnosis and treatment of these infections.

## Abstract 2

Incidence data are lacking, and diagnosis remains difficult.

The >120 recognized species of nontuberculous mycobacteria (NTM) share common features: 1) they are facultative pathogens; 2) evidence of human-to-human transmission is lacking; 3) some NTM species are ubiquitous and others have more restricted distribution; 4) treatment may be difficult and vary according to the involved organism and disease site; and 5) pathogenesis is still undefined, depending on the interaction between the microorganism and the host's immune system (*1*). About 90% of cases involve the pulmonary system; the rest involve lymph nodes, skin, soft tissues, and bones. Less frequently reported are central nervous system disease, keratitis, and otitis media (*1*,*2*). We reviewed the epidemiology, clinical features, diagnosis, and treatment of the most common extrapulmonary diseases associated with NTM in immunocompetent persons (*2*–*5*).

## Lymphadenitis

Localized lymphadenitis most commonly affects children; peak incidence occurs at 1–5 years of age (*6*). The route of infection is hypothesized to be by way of the lymphatic vessels that drain the mouth and pharynx. The most frequently isolated species is *Mycobacterium avium* complex (MAC), followed by *M. scrofulaceum*, *M. malmoense,* and *M. hemophilum* (*7*). However, a growing number of previously unrecognized slow-growing mycobacteria have been implicated with increasing frequency in reports of isolated or microclustered cases (Table 1) (*8*).

**Table 1:** Less frequently encountered mycobacterial species recovered from immunocompetent persons with lymphadenitis*

*Mycobacterium* sp.	No. cases reported	Identification method
*M. bohemicum*	3	GS
*M. celatum*	1	RH, HPLC, GS
*M. genavense*	1	RH, GS
*M. heckeshornense*	1	GS
*M. heidelbergense*	1	GS
*M. interjectum*	4	GS
*M. lentiflavum*	5	HPLC, GS
*M. palustre*	1	GS
*M. parmense*	1	HPLC, GS
*M. simiae*	4	RH, HPLC, GS
*M. triplex*	4	GS
*M. tusciae*	1	HPLC, GS

*Most persons were children. GS, gene sequencing; RH, reverse hybridization; HPLC, high-performance liquid chromatography.

Generally, NTM adenitis is an indolent disease; most patients are otherwise healthy and have as their sole clinical sign a chronic neck mass that does not respond to antimicrobial drug therapy. The disease is usually unilateral and occurs in the cervical, submandibular, or preauricular lymph nodes, although parotid and postauricular node involvement has been reported. The nodes enlarge and may rapidly soften and rupture, forming a draining sinus. Although spontaneous regression has occasionally been described, healing usually occurs by fibrosis and calcification. Pyogenic and tuberculous adenitis are the most important differential diagnoses.

Although a presumptive diagnosis of nontuberculous mycobacterial adenitis can be made on the basis of clinical history and physical examination, definitive diagnosis depends upon the recovery of mycobacteria. Every effort should be made to obtain material for culture and further identification. Cultures of draining and ulcerated lesions, especially when swabs are used for specimen sampling, have been shown to give a lower diagnostic yield than do needle aspirates (*9*) or tissue biopsy samples. For recovery of NTM, use of a liquid medium or radiometric growth detection are regarded as the standard. Moreover, in children who have not been vaccinated with *M. bovis* BCG, purified protein derivative skin testing may be used as a surrogate test to diagnose chronic cervicofacial lymphadenitis (*10*). Histologic appearance of necrotizing granulomatous inflammation with various degrees of caseation is also diagnostic.

Treatment of uncomplicated NTM lymphadenitis is complete surgical excision. Incision and drainage are discouraged because they usually lead to sinus tract formation with chronic discharge. In a recent well-designed trial including 100 children with culture- or PCR-confirmed diagnoses, surgery was more effective than chemotherapy; cure rates were 96% and 66%, respectively (*11*). Total excision should be performed as early as possible to maximize recovery of the causative agent, to prevent further cosmetic damage, to prevent extensive spread and subsequently more difficult excision, and to cure disease. For some patients, however, surgery is associated with substantial risk either because of a discharging sinus or proximity of facial nerve branches. For these patients, chemotherapy preceded by a diagnostic biopsy sample is recommended. Chemotherapy should also be considered for patients in whom lymphadenitis recurs after surgery or for whom all abnormal tissue could not be excised. Recent data indicate fine-needle aspiration as the preferred diagnostic technique for patients with nontuberculous mycobacterial adenitis who do not undergo surgical excision. The optimal chemotherapeutic regimen and its duration are still undetermined, but combination therapy including clarithromycin and a rifamycin, either rifampin or rifabutin and/or ethambutol, may be beneficial.

## Osteoarticular Infections

NTM infections involving the musculoskeletal system are uncommon. However, when they do occur, both rapid- and slow-growing species have been implicated in chronic granulomatous infections involving tendon sheaths, bursae, bones, and joints. These are usually acquired by direct inoculation of the pathogen from an environmental source or a contiguous infection focus as a consequence of surgical procedures, penetrating trauma, injuries, or needle injections. Most affected patients are immunocompetent, but some mycobacterial species, such as *M. chelonae* and *M. hemophilum,* are almost entirely recovered from patients with serious underlying diseases (HIV infection, immunosuppressive therapy, or blood disorders) (*4*). In a recent study of vertebral osteomyelitis caused by NTM (*12*), various degrees of immunosuppression were found in 17 (51.5%) of 33 patients. From the 31 patients with spinal infection studied, the following NTM species were recovered: MAC (n = 13), *M. xenopi* (n = 7), *M. fortuitum* (n = 5), *M. abscessus* (n = 3), *M. kansasii* (n = 1), *M. simiae* (n = 1), and unidentified NTM species (n = 1). *M. hemophilum* also causes NTM osteomyelitis infections in bone marrow and solid organ transplants (*13*). The hand and wrist are the most frequently reported sites of NTM tenosynovitis because of their abundance of synovial fluid and tissue combined with a higher probability of penetrating injury. Most frequently, *M. marinum* and *M. kansasii* are involved; less frequently, *M. avium* complex, *M. szulgai*, *M. terrae*, *M. fortuitum*, *M. chelonae*, *M. abscessus*, *M. malmoense,* and *M. xenopi* are found (*14*).

Clinically, osteoarticular infections caused by NTM are indistinguishable from tuberculosis-associated infections. Signs and symptoms such as localized pain (with or without neurologic impairment), joint stiffness and swelling, low-grade fever, sweating, chills, anorexia, malaise, and weight loss have been reported (Table 2). On rare occasions, suppuration followed by extensive necrosis of the synovial tissue may occur (*3*), although in more severe cases infection may extend to the periosteum and lead to osteomyelitis (*3*). The clinical course of the disease is typically protracted; average time from onset of symptoms to diagnosis may be as long as 10 months. To prevent severe tissue destruction and neurologic disorders, prompt and accurate diagnosis is essential. Diagnosis relies on clinical suspicion and must be considered for patients with increasing musculoskeletal system signs, those with inflammation after penetrating or blunt trauma, and those with underlying risk factors who undergo a medical procedure. Culture of synovial fluid and tissue biopsy are mandatory for definitive diagnosis and identification of the causative agent. Computed tomography and sonography may help guide percutaneous tissue biopsy sampling of infected areas or diagnostic aspiration of intraarticular fluid (*15*). Histopathologic examination has shown a spectrum of inflammatory changes, including granulomatous lesions with or without caseation (*3*).

**Table 2:** Clinical signs associated with osteoarticular infections caused by nontuberculous mycobacteria

Disease	Affected site	*Mycobacterium* spp.	Underlying diseases and risk factors (no. cases reported)
Arthritis, osteomyelitis	Thumb (interphalangeal joint)	*M. malmoense*	Rheumatoid arthritis (1)
Arthritis	Knee, ankle	*M. xenopi*	None* (6), invasive medical procedure (1)
Arthritis	Multiple joints: wrist, knee, finger, ankle, elbow, vertebrae, shoulder	*M. kansasii*	AIDS (13), rheumatoid arthritis (3), systemic lupus erythematous (2), renal transplant (2), polymyositis (1), progressive systemic sclerosis (1), myelodysplasia (1), none (26), localized trauma (10), steroid therapy (10)
Arthritis	Knee	*M. kansasii*	Prosthetic joint (1)
Synovitis (carpal tunnel syndrome)	Wrist	*M. szulgai*	No underlying disease, fish-tank cleaning (2)
Tenosynovitis	Hand	*M. intracellulare*	None* (1)
Tenosynovitis	Hand	*M. chelonae*	Penetrating injury (2), fracture (1), Immunosuppression (3)
Tenosynovitis	Hand, wrist	*M. avium* complex	Steroid injection (1), trauma (8), surgery (4)
Osteomyelitis	Sternum, foot, elbow	*M. wolinsky*	Cardiac surgery (1), stepped on nail (1), open fracture (1)
Osteomyelitis	Femur, tibia, calcaneus, toe, elbow, sternum	*M. goodii*	Open fracture (4), stepped on nail (1), surgery (2), penetrating trauma (2), puncture wound (1)
Osteomyelitis	Wrist	*M. scrofulaceum*	Diabetes (1)
Osteomyelitis	Hand, ankle, wrist	*M. marinum*	Fisherman exposed to aquarium (25), trauma (1), local or systemic steroids (20)
Osteomyelitis		*M. ulcerans*	Traumatic injury (213)
Osteomyelitis	Vertebrae	*M. avium complex, M. xenopi, M. fortuitum, M. abscessus, M. kansasii, M. simiae*	Systemic lupus erythematous and treatment with steroids (7), AIDS (4), interferon receptor defect (3), carcinoma (1), renal failure (1), chronic granulomatous disease (1), none* (16)
Osteomyelitis	Vertebrae	*M. xenopi*	Vertebral disk surgery (58)
Osteomyelitis	Tibia	*M. conceptionense*	Fracture (1)
Osteomyelitis	Vertebrae	*M. abscessus*	Trauma to the back (1)
Osteomyelitis	Vertebrae	*M. avium complex*	Trauma to the back (1)
Osteomyelitis	Lower extremity, upper extremity, vertebrae, disseminated disease	*M. hemophilum*	AIDS (21), bone marrow or solid organ transplant (7); AIDS plus solid organ transplant (1), lymphoma (2), polycythemia vera (1)

*Otherwise healthy with no underlying disease or risk factor.

Lack of the following have greatly limited development of consensus guidelines for the treatment of musculoskeletal infections caused by NTM: correlation between in vitro susceptibility testing and clinical outcome, standardized antimicrobial-drug susceptibility testing for most NTM species, and clinical trials comparing different therapeutic regimens among an adequate number of patients. Prolonged chemotherapy with an isolate-tailored drug combination associated with surgical debridement is currently recommended for all musculoskeletal infections, especially in patients with abscesses (*12*).

## Skin and Soft Tissue Infections

Skin and soft tissue infections usually occur after traumatic injury, surgery, or cosmetic procedures, which may expose a wound to soil, water, or medical devices occasionally contaminated with environmental mycobacteria. Although the epidemiology and clinical presentations of NTM responsible for skin and soft tissue infections differ, some species (MAC, *M. kansasii*, *M. xenopi*, and *M. marinum*) have been reported worldwide, whereas others (*M. ulcerans*) have limited geographic distribution.

*M. marinum* causes diseases in many fish species and is distributed worldwide. It is an opportunistic pathogen of humans, in whom infection is infrequent and occurs by direct injury from fish fins or bites or after cutaneous trauma and subsequent exposure to contaminated water or other sources of infection (shrimp, shellfish, frogs, turtles, dolphin, eels, and oysters). An increasing number of cases have been reported from most countries with temperate climates (*1*). Predisposing occupations and activities include fishery worker, seafood handler, fish-tank owner, fisherman, pet shop worker, and water-related recreational exposure (*4*). Consistent with the organism’s growth at low temperatures, *M. marinum* infections are usually limited to the skin and confined, with few exceptions, to 1 extremity. For fish-tank owners, disease is often located in the hand or fingers; for those who swim in pools, the elbow is affected for 85%, followed by knee and foot (*16*). The incubation period is usually <4 weeks but can be as long as 9 months. Signs and symptoms of early infection are nonspecific, e.g., swelling and pain followed by >1 skin lesions (*17*). At the inoculation site, an erythematous or bluish papulonodular lesion (≈0.5–3 cm) develops and slowly enlarges, becoming more tender until it suppurates (*18*) (Figure). In ≈33% of patients, *M. marinum* infection may spread to deeper structures (soft tissues, tendons, and bone) (*19*), leading to extensive scarring and varying degrees of functional impairment. Less frequently, the disease extends from the inoculation site to regional lymph nodes along the lymphatic vessels, mimicking the clinical appearance of cutaneous sporotrichosis (*20*). Key elements for diagnosis of *M. marinum* infection are a history of exposure to potential sources of infection; a histopathologic appearance of granulomatous inflammation but no caseation; and culture growth of *M. marinum*, which strongly depends on incubation temperature. For localized skin lesions without a history of exposure to fish tanks, swimming pools, or tropical fish, other NTM must be considered (*21*).

**Figure:**
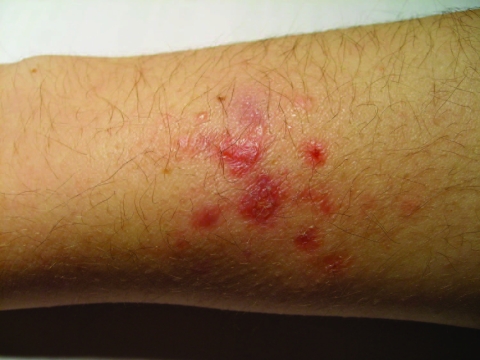
*Mycobacterium marinum* infection of the arm of a fish-tank worker.

Although presumptive identification can rely on a few biochemical and phenotypic tests such as production of photochromogenic pigment, negative nitrate reduction, and positive tests for urease and Tween 80 hydrolysis, definitive identification involves reverse hybridization techniques, or, alternatively, high-performance liquid chromatography analysis of mycolic acids and DNA sequencing assays (*22**,**23*). In vitro susceptibility test results of *M. marinum* clinical isolates have been reported extensively in the literature. Only clarithromycin, minocycline, and amikacin provide complete coverage (100% susceptibility); doxycycline, rifampin, and trimethoprim/sulfamethoxazole encounter different degrees of resistance (*24*). Drug-susceptibility testing of *M. marinum* isolates is recommended only for patients who remain culture positive after several months of therapy (*25*). Although a standardized regimen for *M. marinum* disease is still undefined, monotherapy with doxycycline, minocycline, trimethoprim/sulfamethoxazole, or clarithromycin should be limited to patients with mild disease only. Clarithromycin combined with ethambutol or rifampin is likely the best combination therapy. Treatment with 2 agents should be continued for at least 1–2 months after resolution of skin lesions. In addition, surgical treatment (from mild debridement to amputation) may be required, especially when deep structures are involved (*2*).

*M. ulcerans* is the causative agent of Buruli ulcer, a disease reported in >30 countries, mainly in tropical and subtropical regions of Western and Central Africa but also in Central and South America, Southeast Asia, and the Western Pacific region (*26*). Currently limited knowledge is mainly the result of a low number of reported cases and inadequate surveillance. Recently, however, studies conducted in some areas in which *M. ulcerans* is highly endemic have reported infection rates higher than those for either tuberculosis or leprosy (*27*). Epidemiologic data on detection of *M. ulcerans* DNA in aquatic insects (e.g., orders *Odonata* and *Coleoptera*) and snails, as well as in the biofilm of aquatic plants, strongly suggest that the organism is associated with exposure to surface water involved in environmental changes such as mining, deforestation, agriculture, and hydraulic installations and that it is likely to occupy a specific niche within aquatic environments from which it is transmitted to humans by an unknown mechanism (*28*). It is hypothesized that *M. ulcerans* reaches the human dermis through a cut or wound contaminated with water, soil, or vegetation. *M. ulcerans* is a unique species able to produce a potent, virulence-associated toxin called mycolactone, which prevents phagocytosis of live organisms and induces tissue destruction by its cytotoxic and immunosuppressive properties. Although all age groups can be affected, Buruli ulcer is more frequent among children <15 years of age; the lower limbs (which are involved 3.2× more often than the upper limbs) are the most frequently affected sites (*29*). The incubation period varies but is generally <3 months (*4*). Buruli ulcer usually starts as a single painless subcutaneous nodule or papule, which later moves to form an ulcer with undermined edges (*30*). Spontaneous healing usually takes 4–6 months and involves extensive scar formation, resulting in severe deformity with joint contracture, subluxation, atrophy, or distal lymph edema (*4*). Sometimes tissue destruction may be so extensive that amputation is unavoidable. In addition, dissemination to distant sites can occur, especially in younger patients (<15 years). Multiple lesions represent the most severe form of the disease; a high percentage of cases are osteomyelitis, often leading to amputation or even death (*29*). Disabilities are frequent, estimated for 25% to 58% of cases (*31*). Although in *M. ulcerans*–endemic areas, diagnosis and treatment are determined essentially by clinical appearances, laboratory methods are available. These methods consist of direct smear examination of specimens taken from the ulcer edge or from tissue (≈40% sensitivity); culture incubated at 29°C–33°C for 6–8 weeks (≈20%–60% sensitivity); histopathologic necrosis of subcutaneous tissues and dermal collagen accompanied by a scant nongranulomatous inflammatory reaction embedding acid-fast bacilli (≈90% sensitivity) (*30*); and PCR, a highly sensitive test that can produce results within 2 days but is still confined to reference and research laboratories (*32*). Case confirmation requires at least 2 positive results from the above diagnostic tests (*33*). A standardized method for susceptibility testing of *M. ulcerans* is not currently available (*25*). The main treatment for Buruli ulcer is surgery. To ensure complete removal of visibly affected tissue and to prevent recurrence, excision should include a wide margin, including healthy tissue. Although in early disease simple excision is usually curative, in advanced disease wide and traumatizing excision is needed, followed by skin grafting and long hospital stays. Although many antimicrobial agents have demonstrated excellent in vitro and in vivo activity against *M. ulcerans* clinical isolates, further studies are needed to assess whether experimental susceptibility data will correlate with clinical outcome (*33*). A recent report stated that rifampin plus streptomycin (1×/day for 4 weeks) and surgical excision inhibited the spread of infection and converted early lesions (nodules and plaques) from culture positive to culture negative (*34*). In edematous *M. ulcerans* disease, the most rapidly progressive form, rifampin and streptomycin have demonstrated a dramatically beneficial effect. Other treatments (e.g., topical phenytoin powder, topical nitrogen oxides, and dressing plus triple-drug therapy [rifampin, amikacin, and heparin]) are still being evaluated. *M. bovis* BCG vaccination appears to offer some short-term protection, especially against the most severe form of the disease (*33*).

Cutaneous MAC disease occurs by direct inoculation (trauma, surgery, injection) and is characterized by skin lesions such as ulceration, abscess with sinus formation, or erythematous plaque with a yellow crusted base. The lesions are indolent, with little or no lymph node reaction or systemic symptoms; some MAC skin infections resemble *Lupus vulgaris* infections. Diagnosis requires a high index of suspicion; a history of exposure to a potential source of infection may be suggestive. For an accurate diagnosis, histopathology, proper acid-fast bacilli identification, and susceptibility testing are needed. A combination of excision (or surgical debridement) and chemotherapy is usually required. Therapy is continued for 6–12 months and consists of at least 3 drugs, usually clarithromycin, rifampin, and ethambutol. Additional therapy with amikacin is sometimes included for 6 weeks (*2*).

Rapidly growing mycobacteria (RGM) are a complex group of environmental pigmented and nonpigmented mycobacteria. Their optimal incubation temperatures range from 25°C to 40°C, and they are characterized by a rapid (within 7 days) growth rate on subculture. Organisms responsible for disease in humans belong to the *M. fortuitum* group, the *M. chelonae/abscessus* group, and the *M. smegmatis* group; *M. abscessus*, *M. fortuitum*, and *M. chelonae* are the most common species involved in cutaneous and soft tissue infections. Able to survive in harsh conditions, these organisms produce biofilm in aquatic environments, mostly in piped water systems from which large clumps of mycobacteria are released into water and can be subsequently transmitted to humans (*35*,*36*). In addition, RGM are resistant to sterilizing agents (2% formaldehyde and glutaraldehyde), antiseptics (organomercurial compounds), and other common disinfectants (*37*). Clinical manifestations of RGM disease largely depend on the immunocompetence of the infected person. Cutaneous and soft tissue infections may appear as a single lesion in an immunocompetent person, usually after penetrating trauma or invasive surgical procedure at the site of infection (*M. fortuitum* is the predominant causative agent), or they may appear as multiple or disseminated lesions, usually associated with immunosuppressive treatments (especially long-term treatment with steroids) or other immunosuppressive conditions or concurrent illnesses (*38*). For the latter, *M. chelonae* and *M. abscessus* are the predominant causative agents. Several reported infections that occurred after traumatic, cosmetic, or other medical procedures are summarized in Table 3. In a large outbreak of RGM infection related to pedicures, the median onset of signs and symptoms was 3 weeks; but for some, signs and symptoms were delayed as long as 4 months after exposure (*39*). Histopathologic examination of lesions showed suppurative granulomata and abscesses. Areas of necrosis were typically seen, but caseation was uncommon (*21*). Definitive diagnosis of clinically suspected RGM soft tissue disease can be made by culture of organisms from drainage material, aspiration fluid, or tissue biopsy sample. When NTM disease is suspected on the basis of clinical signs, with any patient history, laboratory staff should be alerted so they can use appropriate isolation protocols (*M. chelonae* and some strains of *M. abscessus* are relatively heat intolerant and can be recovered by primary isolation at 30°C). Identification of RGM at the species level is of utmost importance because treatment regimens and consequently clinical outcome are strongly species related. Proper identification involves molecular techniques (*22*) coupled with a few traditional biochemical and phenotypic tests (use of the carbohydrates citrate, mannitol, inositol, and sorbitol; tolerance to 5% NaCl; nitrate reduction; iron uptake; and 3-day arylsulfatase activity) (*35*). Susceptibility testing performed by the broth microdilution technique (*25*) is essential for choosing the most effective drug therapy and monitoring for the development of mutational drug resistance (*2*). Most experts recommend the use of specific antimicrobial drugs, given in combination to avoid the emergence of drug resistance. Treatment for the *M. fortuitum* group may include amikacin, cefoxitin, ciprofloxacin, the newer quinolones gatifloxacin and moxifloxacin, sulfonamides, and imipenem. Some degree of susceptibility to doxycycline and clarithromycin has been reported. *M. abscessus* strains are usually susceptible to amikacin, cefoxitin, imipenem, clarithromycin, and azithromycin; *M. chelonae* are usually susceptible to amikacin, imipenem, tobramycin, clarithromycin, and sometimes linezolid. Clarithromycin is generally the drug of choice for localized disease caused by *M. chelonae* and *M. abscessus* (*5*,*35*). The duration of therapy is usually 4 months for mild disease and 6 months for severe disease. Surgery is an important complementary tool for treating these infections, depending on disease severity and location.

**Table 3:** Skin and soft tissue infections caused by rapidly growing mycobacteria

Type of infection or procedure	*Mycobacterium* spp.	Clinical findings
Posttraumatic wound infections	*M. fortuitum, M. chelonae, M. abscessus, M. wolinskyi, M. goodii, M. porcinum*	Subcutaneous abscesses, cellulitis
Pedicures	*M. fortuitum, M. chelonae, M. mageritense*	Furunculosis
Subcutaneous, intraarticular, or periarticular Injections	*M. chelonae, M. abscessus*	Subcutaneous abscesses, painful nodules, multiple sinus tracts, joint infections, fever, chills after injection
Acupuncture	*M. abscessus, M. chelonae, M. nonchromogenicum*	Erythematous papules, nodules, ulcerative lesions, abscesses, confluent plaques, draining sinus tracts with discharge, wrist tenosynovitis
Cardiac surgery	*M. peregrinum, M. fortuitum, M. fortuitum* (third biovariant) *complex, M. wolinskyi, M. goodii, M. abscessus*	Sternal wound infection, endocarditis
Cosmetic surgery or other surgical procedures: liposuction, liposculpture, face lift, breast lift (reduction augmentation), silicon injection	*M. fortuitum, M. chelonae, M. chelonae, M. porcinum, M. wolinskyi, M. goodii*	Erythema, tenderness, nodules, skin induration, subcutaneous or deep-tissue abscesses, fever, malaise, multiple abscesses along original suction tracts, draining sinus tracts, local pain, swelling
Nipple piercing	*M. fortuitum, M. abscessus*	Asymptomatic nodules, tender nodules
Implanted with prosthetic material	*M. abscessus, M. goodii*	Abscesses
Pacemaker placement	*M. fortuitum, M. abscessus, M. wolinskyi*	Abscesses
Peritoneal dialysis catheter	*M. abscessus*	Abscesses

## Laboratory Diagnosis

Of all the currently described mycobacterial species, ≈60% have caused human diseases. For this reason, modern techniques for faster culture, identification, and drug susceptibility testing are urgently needed in mycobacteriology laboratories (*22*,*25*). In addition to the collection of high-quality specimens, timely diagnosis of NTM disease requires regular communication of clinical suspicion to the laboratory staff because optimal recover of some fastidious species requires additional tasks. Routine techniques include microscopy and culture; the latter should be performed by using both liquid and solid media incubated at different temperatures (*22*). Although optimal recovery for most clinically relevant mycobacteria is obtained at 35C°–37C°, some species (*M. hemophilum*, *M. marinum, M. ulcerans* and some species of RGM) require a lower incubation temperature to grow. For this reason, all clinical specimens that may harbor the above species (skin, synovial fluid, and bone) should be cultured at 28C°–30C° and at 35C°–37C°. Use of conventional biochemical and phenotypic tests for the identification of NTM is currently discouraged; more rapid and specific methods are favored, including high-performance liquid chromatography analysis of mycolic acids and commercial molecular assays. These may use either in-solution hybridization (Accuprobe, Gen-Probe Inc., San Diego, CA, USA) or solid-format reverse-hybridization assays (line probe assays) (*22*).Both techniques are specific, but the latter (in which amplification precedes hybridization) is more sensitive, enabling identification in the early stage of bacterial growth. Finally, gene (16S rDNA) sequencing is required for those species that cannot be identified by the above systems (*22*). Careful strategies should be recommended for using 16S rDNA sequence analysis databases because public databases may have wrong sequences and commercial ones tend to be underdeveloped and outdated (*23*,*40*).

## Conclusion

Although NTM cause a broad spectrum of human disease, data on incidence of NTM infections are still lacking, mainly because of the absence of systematic epidemiologic studies, standard case definitions, and accurate mycobacterial identification. Furthermore, nonspecific clinical manifestations, lack of familiarity with these infections, and inadequate laboratory services make definitive diagnosis of NTM diseases often delayed or even impossible. Correlation of in vitro susceptibility testing with the clinical outcome, composition and duration of treatment regimens, and use of surgery or other therapeutic approaches are still undefined for most NTM species involved in human diseases. Laboratory research and multicenter controlled trials are needed to improve diagnosis and treatment of extrapulmonary NTM infections.

## References

[1] Falkinham JO. Epidemiology of infection by nontuberculosis mycobacteria. Clin Microbiol Rev. 1996;9:177–215. 8964035 10.1128/cmr.9.2.177 PMC172890

[2] Griffith DE, Aksamit T, Brown-Elliott BA, Catanzaro A, Daley C, Gordin F. An official ATS/IDSA statement: diagnosis, treatment, and prevention of nontuberculous mycobacterial disease. Am J Respir Crit Care Med. 2007;175:367–416. 10.1164/rccm.200604-571ST 17277290

[3] Marchevsky AM, Damsker B, Green S, Tepper S. The clinicopathological spectrum of nontuberculous mycobacterial osteoarticular infections. J Bone Joint Surg Am. 1985;67:925–9. 4019542

[4] Dobos KM, Quinn FD, Ashford DA, Horsburgh CR, King CH. Emergence of a unique group of necrotizing mycobacterial diseases. Emerg Infect Dis. 1999;5:367–78. 10.3201/eid0503.990307 10341173 PMC2640780

[5] De Groote MA, Huitt G. Infections due to rapidly growing mycobacteria. Clin Infect Dis. 2006;42:1756–63. 10.1086/504381 16705584

[6] Lai KK, Stottmeier KD, Sherman IH, McCabe WR. Mycobacterial cervical lymphadenopathy. Relation of etiologic agent to age. JAMA. 1984;251:1286–8. 10.1001/jama.251.10.1286 6422062

[7] Lindeboom JA, Prins JM, Bruijnesteijn van Coppenraet ES, Lindeboom R, Kuijper EJ. Cervicofacial lymphadenitis in children caused by *Mycobacterium haemophilum.* Clin Infect Dis. 2005;41:1569–75. 10.1086/497834 16267728

[8] Tortoli E. Impact of genotypic studies on mycobacterial taxonomy: the new mycobacteria of the 1990s. Clin Microbiol Rev. 2003;16:319–54. 10.1128/CMR.16.2.319-354.2003 12692101 PMC153139

[9] Ellison E, Lapuerta P, Martin SE. Fine needle aspiration diagnosis of mycobacterial lymphadenitis. Sensitivity and predictive value in the United States. Acta Cytol. 1999;43:153–7. 10097702 10.1159/000330969

[10] Lindeboom JA, Kuijper EJ, Prins JM, Bruijnesteijn van Coppenraet ES, Lindeboom R. Tuberculin skin testing is useful in the screening for nontuberculous mycobacterial cervicofacial lymphadenitis in children. Clin Infect Dis. 2006;43:1547–51. 10.1086/509326 17109286

[11] Lindeboom JA, Kuijper EJ, Prins JM, Bruijnesteijn van Coppenraet ES, Lindeboom R, Prins JM. Surgical excision versus antibiotic treatment for nontuberculous mycobacteria cervical lymphadenitis in children: a multicenter, randomized, controlled trial. Clin Infect Dis. 2007;44:1057–64. 10.1086/512675 17366449

[12] Petitjean G, Fluckiger U, Schären S, Laifer G. Vertebral osteomyelitis caused by non-tuberculous mycobacteria. Clin Microbiol Infect. 2004;10:951–3. 10.1111/j.1469-0691.2004.00949.x 15521995

[13] Elsayed S, Read R. *Mycobacterium haemophilum* osteomyelitis: case report and review of the literature. BMC Infect Dis. 2006;6:70. 10.1186/1471-2334-6-70 16606464 PMC1456972

[14] Zenone T, Boibieux A, Tigaud S, Fredenucci JF, Vincent V, Chidiac C, Non-tuberculous mycobacterial tenosynovitis: a review. Scand J Infect Dis. 1999;31:221–8. 10.1080/00365549950163482 10482048

[15] Theodorou DJ, Theodorou SJ, Kakitsubata Y, Sartoris DJ, Resnick D. Imaging characteristics and epidemiological features of atypical mycobacterial infections involving the musculoskeletal system. AJR Am J Roentgenol. 2001;176:341–9. 11159070 10.2214/ajr.176.2.1760341

[16] Casal M, Casal MM; Spanish Group of Mycobacteriology. Multicenter study of incidence of *Mycobacterium marinum* in humans in Spain. [**PMID: 11258515**]. Int J Tuberc Lung Dis. 2001;5:197–9. 11258515

[17] Hess CL, Wolock BS, Murphy MS. *Mycobacterium marinum* infections of the upper extremity. [ **PMID: 15731663**]. Plast Reconstr Surg. 2005;115:55e–9e. 10.1097/01.PRS.0000153197.64808.B9 15731663

[18] Edelstein H. *Mycobacterium marinum* skin infections. Report of 31 cases and review of the literature. Arch Intern Med. 1994;154:1359–64. 10.1001/archinte.154.12.1359 8002687

[19] Aubry A, Chosidow O, Caumes E, Robert J, Cambau E. Sixty-three cases of *Mycobacterium marinum* infection: clinical features, treatment, and antibiotic susceptibility of causative isolates. Arch Intern Med. 2002;162:1746–52. 10.1001/archinte.162.15.1746 12153378

[20] Ang P, Rattana-Apiromyakij N, Goh CL. Retrospective study of *Mycobacterium marinum* skin infections. Int J Dermatol. 2000;39:343–7. 10.1046/j.1365-4362.2000.00916.x 10849123

[21] Bartralot R, Pujol RM, García-Patos V, Sitjas D, Martín-Casabona N, Coll P, Cutaneous infections due to nontuberculous mycobacteria: histopathological review of 28 cases. Comparative study between lesions observed in immunosuppressed patients and normal hosts. J Cutan Pathol. 2000;27:124–9. 10.1034/j.1600-0560.2000.027003124.x 10728814

[22] Clinical and Laboratory Standards Institute. Laboratory detection and identification of mycobacteria: approved guideline. CLSI document M48-A. Wayne (PA): The Institute; 2008.

[23] Cloud JL, Neal H, Rosenberry R, Turenne CY, Jama M, Hillyard DR, Identification of *Mycobacterium* spp. by using a commercial 16S ribosomal DNA sequencing kit and additional sequencing libraries. J Clin Microbiol. 2002;40:400–6. 10.1128/JCM.40.2.400-406.2002 11825949 PMC153382

[24] Bråbäck M, Riesbeck K, Forsgren A. Susceptibilities of *Mycobacterium marinum* to gatifloxacin, gemifloxacin, levofloxacin, linezolid, moxifloxacin, telithromycin, and quinupristin-dalfopristin (Synercid) compared to its susceptibilities to reference macrolides and quinolones. Antimicrob Agents Chemother. 2002;46:1114–6. 10.1128/AAC.46.4.1114-1116.2002 11897601 PMC127113

[25] National Committee for Clinical Laboratory Standards. Susceptibility testing of mycobacteria, nocardia, and other aerobic actinomycetes. Approved standard M24-A. Wayne (PA): The Committee; 2003. 31339680

[26] World Health Organization. Buruli ulcer disease. *Mycobacterium ulcerans* infection: an overview of reported cases globally. Wkly Epidemiol Rec. 2004;79:194–200.15160612

[27] Amofah G, Bonsu F, Tetteh C, Okrah J, Asamoa K. Buruli ulcer in Ghana: results of a national case search. Emerg Infect Dis. 2002;8:167–70. 10.3201/eid0802.010119 11897068 PMC2732443

[28] Marsollier L, Severin T, Aubry J, Merritt RW, Saint Andre JP, Legras P, Aquatic snails, passive hosts of *Mycobacterium ulcerans.* Appl Environ Microbiol. 2004;70:6296–8. 10.1128/AEM.70.10.6296-6298.2004 15466578 PMC522119

[29] Debacker M, Aguiar J, Steunou C, Zinsou C, Meyers WM, Scott JT, *Mycobacterium ulcerans* disease: role of age and gender in incidence and morbidity. Trop Med Int Health. 2004;9:1297–304. 10.1111/j.1365-3156.2004.01339.x 15598261

[30] Guarner J, Bartlett J, Whitney EA, Raghunathan PL, Stienstra Y, Asamoa K, Histopathologic features of *Mycobacterium ulcerans* infection. Emerg Infect Dis. 2003;9:651–6. 12780997 10.3201/eid0906.020485 PMC3000137

[31] Ellen DE, Stienstra Y, Teelken MA, Dijkstra PU, van der Graaf WT, van der Werf TS. Assessment of functional limitations caused by *Mycobacterium ulcerans* infections: towards a Buruli ulcer functional limitation score. Trop Med Int Health. 2003;8:90–6. 10.1046/j.1365-3156.2003.00976.x 12535257

[32] Ross BC, Marino L, Oppedisano F, Edwards R, Robins-Browne RM, Johnson PD. Development of a PCR assay for rapid diagnosis of *Mycobacterium ulcerans* infection. J Clin Microbiol. 1997;35:1696–700. 9196176 10.1128/jcm.35.7.1696-1700.1997 PMC229824

[33] Wansbrough-Jones M, Phillips R. Buruli ulcer: emerging from obscurity. Lancet. 2006;367:1849–58. 10.1016/S0140-6736(06)68807-7 16753488

[34] Etuaful S, Carbonnelle B, Grosset J, Lucas S, Horsfield C, Phillips R, Efficacy of the combination rifampin-streptomycin in preventing growth of *Mycobacterium ulcerans* in early lesions of Buruli ulcer in humans. Antimicrob Agents Chemother. 2005;49:3182–6. 10.1128/AAC.49.8.3182-3186.2005 16048922 PMC1196249

[35] Brown-Elliott BA, Wallace RJ Jr. Clinical and taxonomic status of pathogenic nonpigmented late-pigmenting rapidly growing mycobacteria. Clin Microbiol Rev. 2002;15:716–46. 10.1128/CMR.15.4.716-746.2002 12364376 PMC126856

[36] Hall-Stoodley L, Stoodley P. Biofilm formation and dispersal and the transmission of human pathogens. Trends Microbiol. 2005;13:7–10. 10.1016/j.tim.2004.11.004 15639625

[37] Wallace RJ Jr, Brown BA, Griffith DE. Nosocomial outbreaks/pseudo-outbreaks caused by nontuberculous mycobacteria. Annu Rev Microbiol. 1998;52:453–90. 10.1146/annurev.micro.52.1.453 9891805

[38] Uslan DZ, Kowalski TJ, Wengenack LW, Virk A, Wilson JW. Skin and soft tissue infections due to rapidly growing mycobacteria. Arch Dermatol. 2006;142:1287–92. 10.1001/archderm.142.10.1287 17043183

[39] Winthrop KL, Abrams M, Yakrus M, Schwartz I, Ely J, Gillies D, An outbreak of mycobacterial furunculosis associated with footbaths at a nail salon. N Engl J Med. 2002;346:1366–71. 10.1056/NEJMoa012643 11986410

[40] Turenne CY, Tschetter L, Wolfe J, Kabani A. Necessity of quality-controlled 16S rRNA gene sequence databases: identifying nontuberculous *Mycobacterium* species. J Clin Microbiol. 2001;39:3637–48. 10.1128/JCM.39.10.3638-3648.2001 11574585 PMC88401

